# Multiple cancer site comparison of adjusted survival by hospital of treatment: an East Anglian study

**DOI:** 10.1054/bjoc.1999.0901

**Published:** 1999-12-08

**Authors:** D Stockton, T Davies

**Affiliations:** East Anglian Cancer Intelligence Unit,niversity of Cambridge, Strangeways Research Laboratory,ambridge, UK; East Anglian Cancer Intelligence Unit,niversity of Cambridge, Institute of Public Health,obinson Way, Cambridge, CB2 2SR, UK

**Keywords:** survival, Calman–Hine, cancer, hospital, tumour stage

## Abstract

We performed a preliminary investigation into which hospitals would benefit frominvestment and development, and which should have services restricted, with respect to the implementation of the Calman–Hine strategy of specialist cancer care. A retrospective study approach was used implementing uniform definitions for colon, rectal, breast, melanoma, bladder and ovarian cancers. A total of 14 527 cases registered by the East Anglian cancer registry and diagnosed between 1989 and 1993 were included. The cases were analysed in two age groups (< 75, 75+ years) and two hospital groups: group 1, those treated at hospitals with radiotherapy and oncology departments; group 2, other district general hospitals. Adjusted hazard ratios derived from Cox's proportional hazards model and adjusted conditional survival curves were presented. We found that afterdjustment for age, sex and tumour stage at diagnosis, survival up to 5 years after diagnosis was usually worse in group 2 hospitals and significantly so for patients aged < 75 years with breast, ovarian and rectal tumours. Hospital workload produced little significant effect independently from hospital group. Analysing the selected cancer sites using uniform definitions and consistent staging supports the view that the strategy proposed in the Calman–Hine report is likely to be beneficial, but particular priority for change should be given to younger patients with breast, ovarian and rectal tumours. © 2000 Cancer Research Campaign


*British Journal of Cancer* (2000) **82**(1), 208–212
© 2000 Cancer Research Campaign
Article no. bjoc.1999.0901

### Multiple cancer site comparison of adjusted survival by hospital of treatment: an East Anglian study

**D Stockton[1] and T Davies[2]**

[1]East Anglian Cancer Intelligence Unit, University of Cambridge, Strangeways Research Laboratory, Cambridge, UK; [2]East Anglian Cancer Intelligence Unit, University of Cambridge, Institute of Public Health, Robinson Way, Cambridge CB2 2SR, UK

**Summary** We performed a preliminary investigation into which hospitals would benefit from investment and development, and which should have services restricted, with respect to the implementation of the Calman–Hine strategy of specialist cancer care. A retrospective study approach was used implementing uniform definitions for colon, rectal, breast, melanoma, bladder and ovarian cancers. A total of 14 527 cases registered by the East Anglian cancer registry and diagnosed between 1989 and 1993 were included. The cases were analysed in two age groups (< 75, 75+ years) and two hospital groups: group 1, those treated at hospitals with radiotherapy and oncology departments; group 2, other district general hospitals. Adjusted hazard ratios derived from Cox's proportional hazards model and adjusted conditional survival curves were presented. We found that after adjustment for age, sex and tumour stage at diagnosis, survival up to 5 years after diagnosis was usually worse in group 2 hospitals and significantly so for patients aged < 75 years with breast, ovarian and rectal tumours. Hospital workload produced little significant effect independently from hospital group. Analysing the selected cancer sites using uniform definitions and consistent staging supports the view that the strategy proposed in the Calman–Hine report is likely to be beneficial, but particular priority for change should be given to younger patients with breast, ovarian and rectal tumours. © 2000 Cancer Research Campaign

**Keywords:** survival; Calman–Hine; cancer; hospital; tumour stage

The strategy put forward in the Calman–Hine report 'A policy framework for commissioning cancer services' (Department of Health, 1994) proposes that patients with cancer should be treated only in hospitals with enough staff, resources and collective expertise for optimum treatment of the specific cancer. The report relied, in part, on evidence that the best care for cancer patients depended on having a team of specialists and a minimum workload for each specialist that ensured that they maintained and developed their expertise. In general terms this meant that the expected pattern of development of cancer services would result in cancer units, mainly in district general hospitals, which would have sufficient workload to maintain expertise in treating commoner cancers, and cancer centres which, while performing the functions of a unit, would treat the less common cancers, and would also act as a source of specialization not usually found in a district general hospital.

Implementation of this policy means that health authorities have to alter the present pattern of services, which means they need to know which hospitals would benefit from more investment and development, and which should have services restricted or altered by pooling their resources with other hospitals.

In the Anglia and Oxford region this process started by establishing the current workload of hospitals, and at the same time a series of meetings were held involving clinical staff with the aim of deciding what kinds of services should be provided for which cancers. The Anglia and Oxford NHS Executive initially have considered lung, gastrointestinal, breast, skin, urological cancers, gynaecological cancers, brain and central nervous system, haematological cancer, children's cancers and head and neck cancers. The health authorities had expressed a particular interest in the performance of individual hospitals.

While there are many aspects of hospital performance, the East Anglian Cancer Registry is able to provide information on patient survival by individual hospital as they collect information on tumour stage at diagnosis and perform active follow-up of patients, thus ensuring accurate survival information with the added ability to adjust for case mix differences. This allows uniform analyses over different cancer sites to be performed, so that groups of patients for whom implementation of the Calman–Hine report will be the most effective (or not effective) can be identified.

### MATERIALS AND METHODS

All invasive cancers for the sites fitting our inclusion criteria described below, diagnosed between 1989 and 1993 (to allow survival analyses to be performed for patients followed up until the end of 1998) and registered by the East Anglian Cancer Registry were identified. The inclusion criterion was defined as all cancer sites being considered by the Anglia and Oxford NHS Executive for which the registry has an adequate (at least 70%) proportion of cancers staged over the period. Thus we included colon, rectal, breast, melanoma, bladder and ovarian cancers. Active follow-up of patients is carried out by the Registry at regular intervals (3 years after diagnosis, 5 years after diagnosis and then every 5 years until death), so vital status 3 years after diagnosis was known for almost all (98.7%) of the 16 367 patients identified, and after 5 years for 80.4% of patients. Patients with

*Received 2 November 1998*
*Revised 8 June 1999*
*Accepted 8 June 1999*

*Correspondence to:* T Davies

**Table 1** Patients diagnosed 1989–1993: number of cases and percentage seen at group 1 hospitals by tumour type, hospital group and age stratum

| Diagnosis | Age group | Group 1 (3 hospitals) | Group 2 (6 hospitals) | Total cases |
|---|---|---|---|---|
| Breast | <75 | 2093 (49%) | 2220 | 4313 |
| | 75+ | 648 (51%) | 615 | 1263 |
| | Total | 2741 (49%) | 2635 | 5576 |
| Ovary | <75 | 336 (47%) | 383 | 719 |
| | 75+ | 139 (51%) | 131 | 270 |
| | Total | 475 (48%) | 514 | 989 |
| Colon | <75 | 907 (46%) | 1045 | 1952 |
| | 75+ | 750 (46%) | 898 | 1648 |
| | Total | 1657 (46%) | 1943 | 3600 |
| Rectal | <75 | 553 (44%) | 691 | 1244 |
| | 75+ | 353 (45%) | 426 | 799 |
| | Total | 906 (45%) | 1117 | 2023 |
| Bladder | <75 | 401 (51%) | 377 | 778 |
| | 75+ | 428 (58%) | 307 | 735 |
| | Total | 829 (55%) | 684 | 1513 |
| Melanoma | <75 | 319 (49%) | 377 | 656 |
| | 75+ | 73 (43%) | 97 | 170 |
| | Total | 392 (47%) | 434 | 826 |

fewer than 5 years of follow-up available, or those lost to follow-up, were entered into the analysis as censored cases. All registrations are reviewed and, where possible, staged by the medical director (a clinical oncologist) of the registry, thus ensuring consistency of staging in the population over the study period. Cases were staged according to the TNM classification of malignant tumours (Hermanek and Sobin, 1987) using all the staging information available including clinical and pathological information. Some patients, especially the elderly, did not have enough information in the records for staging (see Table 2). These patients are included in the analysis as 'unstaged'.

For local purposes, the data were initially analysed by individual hospital (results not presented here). The data were then grouped so that hospitals with radiotherapy and oncology departments (Addenbrooke's in Cambridge, the Norfolk and Norwich Hospital, and Ipswich Hospital) [group 1] (7000 patients) could be compared with the six district general hospitals without radiotherapy and oncology departments [group 2] (7527 patients). Hospitals that are not district general hospitals are usually small and are mostly community hospitals dealing with small numbers of patients. In East Anglia there are five such hospitals, including one private hospital. Patients attending these, along with other privately treated and extra regionally treated patients, were excluded from the analysis because the numbers were too small to form meaningful groups of their own. In total they covered 11% (1840 patients) of the potential study population. If a patient attended more than one hospital then they were assigned to the hospital where the primary treatment was delivered.

Cox's proportional hazards regression models (Cox, 1972) were analysed to investigate survival differences for patients treated at group 1 compared to group 2 hospitals adjusting for sex, age (in 10-year age bands) and tumour stage at diagnosis. As the group 1 hospitals were usually hospitals treating large numbers of patients (high workload hospitals), the relative importance of specialization and hospital workload was also investigated using Cox's proportional hazards regression. Sub-analyses were performed to compare the data both adjusted and unadjusted for tumour stage, and in two age groups: under 75 and 75+ years, under the assumption that aggressive curative treatment attempts would be more likely in younger patients.

For the sub-groups where significant differences in hazard were found, survival curves were produced using adjusted conditional probabilities (Nieto and Coresh, 1996), with adjustment for the covariates sex, age (in 10-year age bands) and tumour stage. These adjusted conditional probabilities illustrate the survival we would expect in the group 2 hospital patients if they had the same covariate composition as the group 1 hospital patients.

**Table 2** Patients diagnosed 1989–1993: percentage of patients presenting at each TNM tumour stage (UICC classification) at diagnosis by tumour type and age stratum. Hospital groups are shown separately: % in group 1 first and then % in group 2 in brackets.

| Diagnosis | Age group | % Stage 1 | % Stage 2 | % Stage 3 | % Stage 4 | % Not staged |
|---|---|---|---|---|---|---|
| Breast | <75 | 43% (44%) | 42% (39%) | 7% (7%) | 5% (6%) | 4% (3%) |
| | 75+ | 18% (21%) | 37% (32%) | 17% (15%) | 9% (10%) | 19% (22%) |
| | Total | 37% (39%) | 41% (38%) | 10% (9%) | 6% (7%) | 7% (7%) |
| Ovary | <75 | 23% (28%) | 14% (11%) | 41% (36%) | 11% (12%) | 12% (13%) |
| | 75+ | 12% (12%) | 9% (10%) | 36% (28%) | 13% (12%) | 30% (39%) |
| | Total | 20% (24%) | 13% (11%) | 39% (34%) | 11% (12%) | 17% (20%) |
| Colon | <75 | 8% (7%) | 37% (38%) | 28% (30%) | 18% (17%) | 9% (8%) |
| | 75+ | 7% (6%) | 35% (36%) | 22% (25%) | 16% (13%) | 20% (21%) |
| | Total | 8% (7%) | 36% (37%) | 25% (28%) | 17% (15%) | 14% (14%) |
| Rectal | <75 | 20% (15%) | 26% (32%) | 28% (28%) | 16% (12%) | 11% (12%) |
| | 75+ | 18% (15%) | 27% (26%) | 18% (19%) | 12% (11%) | 24% (30%) |
| | Total | 19% (15%) | 26% (30%) | 25% (25%) | 14% (12%) | 16% (19%) |
| Bladder | <75 | 37% (44%) | 23% (17%) | 14% (18%) | 6% (7%) | 20% (13%) |
| | 75+ | 35% (38%) | 24% (15%) | 15% (16%) | 7% (8%) | 19% (24%) |
| | Total | 36% (41%) | 23% (16%) | 15% (17%) | 7% (7%) | 20% (18%) |
| Melanoma | <75 | 46% (49%) | 23% (17%) | 9% (11%) | 5% (5%) | 18% (18%) |
| | 75+ | 21% (23%) | 36% (20%) | 25% (28%) | 3% (5%) | 16% (25%) |
| | Total | 41% (43%) | 25% (18%) | 12% (15%) | 5% (5%) | 17% (20%) |

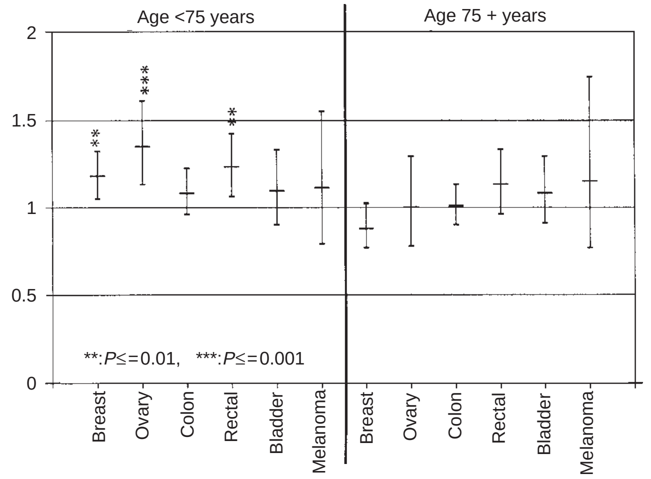

**Figure 1** Hazard ratios comparing survival up to 5 years after diagnosis in group 2 relative to Group 1 hospitals by tumour and age group. Adjusted for 10-year age band, sex and stage at diagnosis

### RESULTS

A total of 14 527 patients were included in the final analysis. The numbers of cancers by tumour type, age stratum and hospital group are shown in Table 1. The group 1 hospitals treated between 45% (rectal) and 55% (bladder) of all cases and saw a similar proportion of those aged under 75 years and those aged 75+ years. Table 2 shows the percentage of cancers presenting in each tumour stage within hospital group and age stratum. The stage distribution for group 1 and group 2 hospitals was very similar.

Cox's proportional hazards ratios comparing group 2 with group 1 hospitals with adjustment for age (in 10-year age bands) and sex are shown in Table 3. Survival up to 5 years after diagnosis was generally worse in group 2 hospitals and significantly so for rectal tumours (model I). This difference was accentuated when an adjustment for tumour stage was added to the model (model II) indicating that survival within stage was worse in group 2 hospitals, particularly for rectal and ovarian tumours.

Cox's proportional hazards ratios comparing group 2 with group 1 hospitals calculated separately for each age group with adjustment for age (within the age group), sex and tumour stage are shown in Figure 1. For patients aged under 75 years, survival up to 5 years after diagnosis was worse in group 2 hospitals for the tumour types and significantly so for breast, ovarian and rectal tumours. For older patients, the trend of worse survival in group 2 hospitals was also evident except for breast cancer. The differences in older patients were not significant.

Figure 2A–C show plots of survival up to 5 years after diagnosis in group 1 and 2 hospitals for breast, ovary and rectal tumours for patients aged less than 75 years. The crude Kaplan–Meier survival for group 1 and group 2 hospitals is plotted along with the adjusted conditional probability of the survival we would expect in the group 2 hospital patients if they had the same covariate composition as the group 1 hospital patients.

Hospital workload and hospital groups are not independent, since the hospitals with radiotherapy and oncology departments tend to have a larger workload. In order to distinguish the effects of workload we analysed (the logarithm of) the individual hospital workloads. Group 1 and group 2 hospitals were analysed separately because the group 1 hospitals are almost always the hospitals with the largest workload making a stratified analysis meaningless. The hazard ratios shown in Table 4 suggest that workload produces little significant effect independently from hospital group, except for melanoma skin cancer at group 1 hospitals where an inverse effect is seen.

**Table 3** Hazard ratios comparing survival up to 5 years after diagnosis in Group 2 relative to Group 1 hospitals by tumour type

| Diagnosis | Model I | Model II |
|---|---|---|
| Breast | 1.03 (0.94–1.13) | 1.04 (0.95–1.14) |
| Ovary | 1.12 (0.97–1.30) | 1.17 (1.01–1.35)[a] |
| Colon | 0.98 (0.91–1.06) | 1.04 (0.96–1.13) |
| Rectal | 1.12 (1.01–1.25)[a] | 1.19 (1.06–1.32)[b] |
| Bladder | 1.09 (0.96–1.24) | 1.09 (0.96–1.24) |
| Melanoma | 1.13 (0.88–1.46) | 1.17 (0.91–1.50) |

[a]$P \leq 0.05$, [b]$P \leq 0.01$, [c]$P \leq 0.001$. Adjusted for (I) 10-year age band and sex and (II) 10-year age band, sex and TNM tumour stage (UICC classification) at diagnosis.

**Table 4** Hazard ratios comparing survival up to 5 years after diagnosis using the logarithms of the individual hospital workloads, by tumour type and hospital group

| Hospital group | Diagnosis | Hazard ratio | 95% confidence interval |
|---|---|---|---|
| Group 1 | Breast | 0.91 | 0.67–1.24 |
| | Ovary | 1.48 | 0.79–2.74 |
| | Colon | 1.20 | 0.78–1.85 |
| | Rectal | 0.98 | 0.63–1.54 |
| | Bladder | 0.81 | 0.57–1.16 |
| | Melanoma | 1.80 | 1.06–3.06[a] |
| Group 2 | Breast | 1.07 | 0.93–1.23 |
| | Ovary | 1.03 | 0.83–1.30 |
| | Colon | 0.94 | 0.79–1.26 |
| | Rectal | 0.97 | 0.80–1.19 |
| | Bladder | 0.96 | 0.80–1.15 |
| | Melanoma | 1.03 | 0.75–1.41 |

[a]$P \leq 0.05$. Adjusted for 10-year age band, sex and TNM tumour stage (UICC classification) at diagnosis.

### DISCUSSION

For the patients included in this study, survival up to 5 years after diagnosis was significantly worse for patients with ovarian, rectal and breast tumours if they were aged under 75 years at diagnosis, and had their main treatment in hospitals without radiotherapy and oncology departments. These hazards have been adjusted for case mix in terms of year of age, sex (where appropriate) and TNM tumour stage at diagnosis. The differences in hazard can be seen clearly in the survival graphs (see Figure 2).

### Stage migration

The East Anglian Cancer Registry has very good information on staging, and this has allowed us to use stage as a means of adjusting for case mix. Nevertheless, staging is not complete, and

© 2000 Cancer Research Campaign

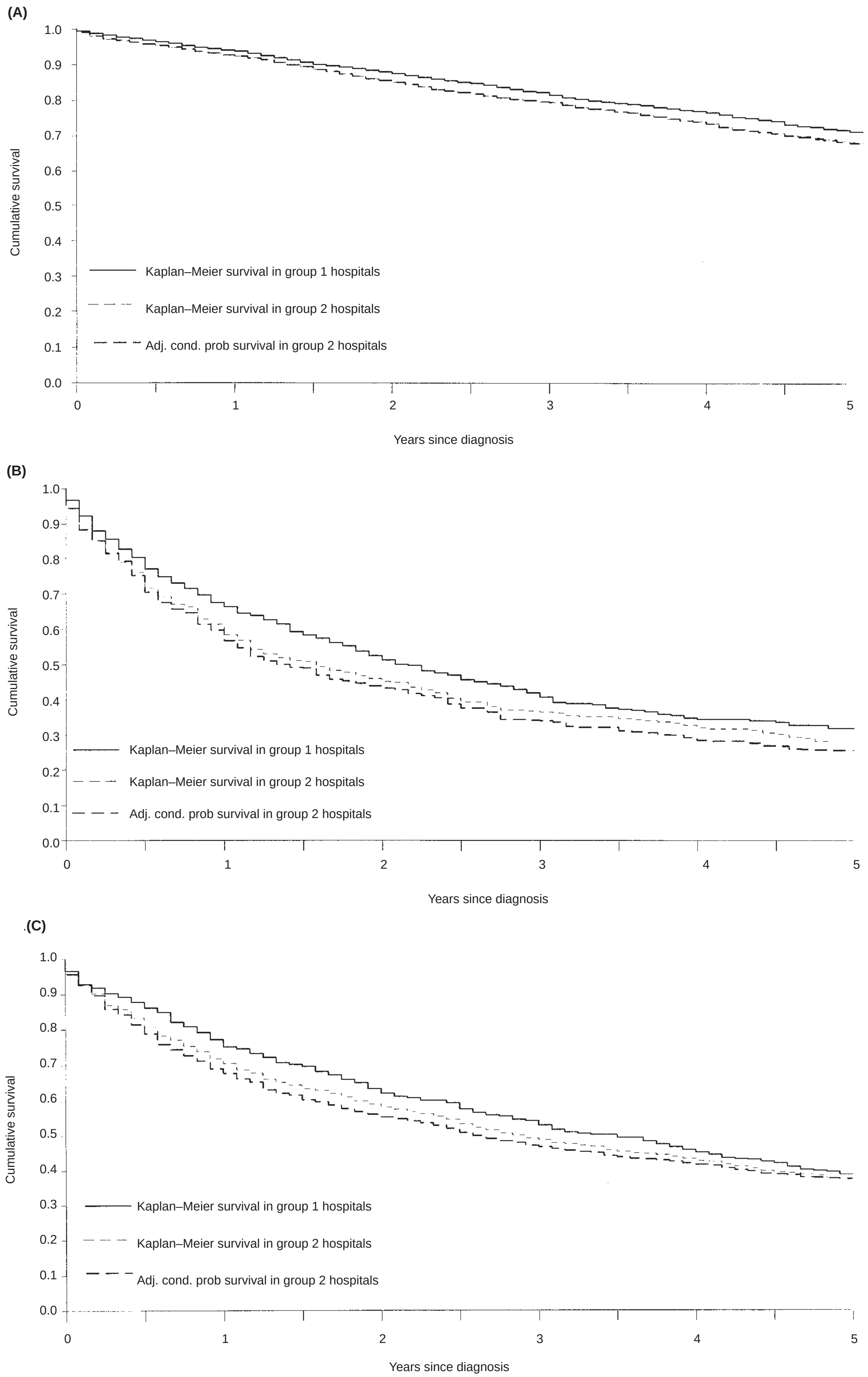

**Figure 2** Survival rates for patients < 75 years: (**A**) breast cancer, (**B**) ovarian cancer, (**C**) rectal cancer the accuracy of staging and completeness of staging could influence the results since misclassification results in stage migration. For example, if staging of all tumours at a certain site in a hospital is subject to bias and they are given an earlier stage classification than they should have, then survival becomes apparently worse in the earlier stage to which tumours are allocated, and in the more advanced stage from which they have been withdrawn. Thus a hospital with poorly reported histology or poor imaging is not only likely to give patients inappropriate treatment but also bias its own stage-adjusted results to make them look worse still. We found one hospital where this appeared to have happened (in the reporting of bladder cancers), but grouping of hospitals is likely to make this effect small. The analysis in Table 3 indicates a survival advantage for patients treated at group 1 hospitals even without an adjustment for tumour stage, although it is not as marked as the stage-adjusted analysis, which could indicate stage migration or within stage survival variation.

### Missing stage

Staging of cancers in East Anglia is supervised by the Medical Director (a clinical oncologist) of the Registry and is thus likely to be uniform, though still depending on adequate information. Where this was not available, a tumour was classified as unstaged. If a large number of tumours were unstaged (indicating complete data unavailable), 'unstaged' would come to represent the average tumour with a better survival than unstaged tumours where staging was not possible because the patient was too ill. Hospitals where this happened would thus have a contribution from an apparently relatively good survival in unstaged patients and thus, in adjusting for stage, their overall survival would improve because the analysis treats 'unstaged' as quantitatively equivalent to true stages. This was observed in one hospital where staging information for 60% of one tumour was omitted by mistake with the predicted effect. This was put right. The range of the proportion of the remaining unstaged tumours is fairly small and, given the pooling of hospital results, unlikely to affect estimates of mortality hazard.

The reason for the survival advantage in specialist hospitals being more evident in patients aged less than 75 years is not clear. Survival as measured here takes into account age, sex and tumour stage, but does not allow for differences in all cause mortality, i.e. death from causes other than cancer will also have influence in the survival figures. For elderly patients all cause mortality will clearly be higher and it could be that this effect masks the differences seen between hospitals in the younger age groups. On the other hand, it might be that the factors that are associated with improved survival in younger patients are not applied to elderly patients. A third possibility is that the smaller district general hospitals are relatively better at treating elderly patients.

Another interpretation for the improved survival in specialist hospitals in patients aged less than 75 years is that patients with a better prognosis may be selectively referred to the group 1 hospitals. This would leave an excess of poor prognosis and elderly patients at the group 2 hospitals and thus make the survival appear artificially worse. Conversely, the specialist hospitals may receive advanced cases that require complicated treatment regimes making survival appear worse. We have found no evidence that either of these situations were systematically occurring (see Table 2), or if they were, that they would be specific to patients diagnosed with ovarian, breast or rectal cancer. Adjusting for age and stage at diagnosis should also help eliminate any bias of this kind.

Selby et al (1996) recently published a review on papers looking at outcome in terms of survival. From this review most of the evidence suggests that there is usually some benefit from specialized cancer care and this would apply to treatment of tumours of the breast, ovary, colon and rectum. However, it is inevitable that evidence from many different studies is not consistent in definitions of specialization and case mix, and that the information available on variables is likely to affect apparent outcome. The study reported in this paper, using routinely collected cancer registration data, has uniform definitions and it is clear which tumours are more successfully treated in specialized hospitals, taking into account stage as the indicator of case mix.

The initial purpose of the analysis was to assess the performance of individual hospitals. There was wide variation that probably reflected differences in case mix (not taken into account by stage), random variation and the performance of one or two individuals. However, when the results are pooled, these variations must lose their impact; in particular, geographic confounders since the catchment areas of the pooled hospitals overlap. The implication is that there is a real difference, in this region and this period in the performance of specialized and non-specialized hospitals for certain tumours in patients aged under 75 years. This does not simply appear to be due to bigger hospital workload for the tumour site. For colon and rectal cancers there is evidence of more chemotherapy being used for patients treated at the group 1 hospitals over the study period which should be investigated further, but the question of why these differences are seen cannot really be answered with the routine data we have available.

The health authorities who have received this information are naturally hesitant to take dramatic action based on this information alone, but nevertheless it does lend more support to the view that the strategy proposed in the Calman–Hine report is likely to be beneficial.

### ACKNOWLEDGEMENTS

We would like to thank Mrs Marjorie Page and her staff at the East Anglian cancer registry for their invaluable work. We would also like to thank Dr David Speigalhalter and Dr Peter Treasure for their comments during the preparation of this paper, and one of the anonymous referees whose comments greatly improved this paper.

### REFERENCES

Cox DR (1972) Regression models and life tables. *J R Stat Soc* **34**: 187–220

Department of Health (1994) Consultative document: a policy framework for commissioning cancer services. Department of Health: London

Hermanek P and Sobin LH (ed) (1987) UICC International Union Against Cancer. *TNM Classification of Malignant Tumours*, 4th edn. Springer-Verlag: Berlin

Nieto FJ and Coresh J (1996) Adjusting survival curves for confounders: a review and a new method. *Am J Epidemiol* **143**: 1059–1068

Selby P, Gillis C and Haward R (1996) Benefits from specialised cancer care. *Lancet* **348**: 313–318

© 2000 Cancer Research Campaign
